# Scaling of Convex Hull Volume to Body Mass in Modern Primates, Non-Primate Mammals and Birds

**DOI:** 10.1371/journal.pone.0091691

**Published:** 2014-03-11

**Authors:** Charlotte A. Brassey, William I. Sellers

**Affiliations:** Faculty of Life Sciences, University of Manchester, Manchester, United Kingdom; Monash University, Australia

## Abstract

The volumetric method of ‘convex hulling’ has recently been put forward as a mass prediction technique for fossil vertebrates. Convex hulling involves the calculation of minimum convex hull volumes (*vol*_CH_) from the complete mounted skeletons of modern museum specimens, which are subsequently regressed against body mass (*M*_b_) to derive predictive equations for extinct species. The convex hulling technique has recently been applied to estimate body mass in giant sauropods and fossil ratites, however the biomechanical signal contained within *vol*_CH_ has remained unclear. Specifically, when *vol*_CH_ scaling departs from isometry in a group of vertebrates, how might this be interpreted? Here we derive predictive equations for primates, non-primate mammals and birds and compare the scaling behaviour of *M*_b_ to *vol*_CH_ between groups. We find predictive equations to be characterised by extremely high correlation coefficients (*r* ^2^ = 0.97–0.99) and low mean percentage prediction error (11–20%). Results suggest non-primate mammals scale body mass to *vol*_CH_ isometrically (*b* = 0.92, 95%CI = 0.85–1.00, *p* = 0.08). Birds scale body mass to *vol*_CH_ with negative allometry (*b* = 0.81, 95%CI = 0.70–0.91, *p* = 0.011) and apparent density (*vol*_CH_/*M*_b_) therefore decreases with mass (*r* ^2^ = 0.36, *p*<0.05). In contrast, primates scale body mass to *vol*_CH_ with positive allometry (*b* = 1.07, 95%CI = 1.01–1.12, *p* = 0.05) and apparent density therefore increases with size (*r* ^2^ = 0.46, *p* = 0.025). We interpret such departures from isometry in the context of the ‘missing mass’ of soft tissues that are excluded from the convex hulling process. We conclude that the convex hulling technique can be justifiably applied to the fossil record when a large proportion of the skeleton is preserved. However we emphasise the need for future studies to quantify interspecific variation in the distribution of soft tissues such as muscle, integument and body fat.

## Introduction

An animal’s form and function is bound by physical laws. They determine the strength of structures, the rate of heat transfer and the dynamics of locomotion [1], and their consequences are dependent upon the mass of the body on which they act. As such, an organism’s mass is a critical constraint on its growth, physiology, ecology and biomechanics. Quantitative predictions of the mass properties of extinct taxa are therefore crucial to understanding their palaeobiology, and considerable effort has gone into deriving such mass estimates.

Common practice when estimating fossil body mass has been to take a skeletal dimension from modern species, such as femur circumference [2] or glenoid diameter [3], and use this value as the independent variable in a regression against body mass [4]. This method has been subject to considerable discussion in the literature and concerns have been raised regarding logarithmic transformation of the dataset [5], the choice of regression model [6] and the extrapolation of the model beyond the range of extant data [7]. Bivariate regressions also suffer from the ‘single bone problem’, in which reliance upon a single metric derived from a highly specialised skeletal element to predict body mass may result in considerable over- or underestimation [8]. When only fragmentary material is preserved, however, this remains the only available method for predicting body mass of extinct species.

In contrast, volumetric techniques require a reconstruction of the entire skeleton and do not rely upon single skeletal elements for mass estimation. Early attempts at volumetric reconstructions involved the construction of physical scale models and estimates of fluid displacement [9]–[11]. More recently, digital models have been created with the purpose of estimating mass and inertial properties of individual body segments [12]–[17]. In these instances, 3D mathematical slices may be fitted to given frontal and sagittal profiles [12], [13], B-spline objects can be fitted to control points on the skeleton [14], [15] or a single continuous surface may be lofted between several B-spline curves [16], [17]. The digital models can then be ‘fleshed out’ to reflect body contours *in vivo*. In these studies, the authors reflect upon the issues associated with ‘artistic’ modelling of fossil body shape, and carry out sensitivity analyses in order to quantify the effect of soft tissue reconstructions on mass estimates. Furthermore, in order to estimate mass, a value for body density (ρ_b_) must be assigned to the volumetric model. Values for body density are sparsely reported in the literature (see discussion and Table S1 for more detail) and in the case of fossil species, a value of 1000–1024 kg/m^−3^ (the density of water) is often assigned [11]–[17]. Additional inferences must then be made regarding the size and location of air-filled cavities such as lungs and air sacs.

**Figure 1:**
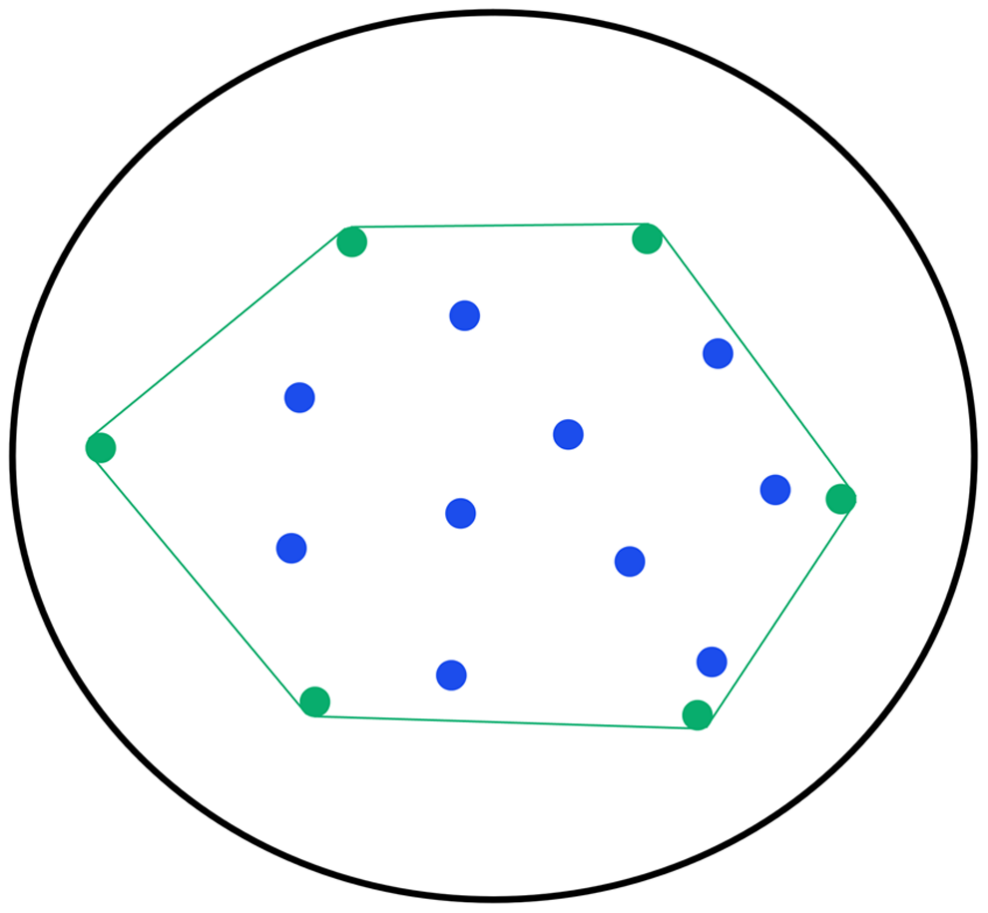
Simplified example of the convex hulling process. Black ellipse represents the initial extent of a rubber band stretched to encompass all coloured points. Green polygon represents the convex hull defined by the rubber band ‘snapping to’ the green boundary points. The internal (blue) points lie within the convex hull and do not contribute to defining its maximum extent.

Convex hulling is an alternative approach to body mass estimation that has recently been put forward, which combines aspects of both volumetric modelling and linear regression [8], [18]. Much the same as other volumetric techniques, convex hulling benefits from including the maximum amount of information from the skeleton into the mass estimate and circumvents the ‘single bone problem’ compared with regressions based on isolated limb bone dimensions. Convex hulling also sidesteps the requirements for soft tissue reconstructions that are necessary in other volumetric mass estimates, and has been applied to estimate fossil body mass in two species of giant bird [8] and sauropod dinosaurs [18], [19]. The convex hull (*CH*) is one of the oldest and most important structures within the field of computational geometry. The convex hull *CH(S)* of set of points *S* is the smallest convex polytope that contains *S* [20] (Figure 1), which, intuitively in 2D, can be thought of as stretching a rubber band around a given set of points. The practical application of calculating convex hulls has ranged from determining trait space in biotic community assemblages [21] to collision detection in computer games design [22] and solving shortest-path problems in transport logistics [23].

**Figure 2:**
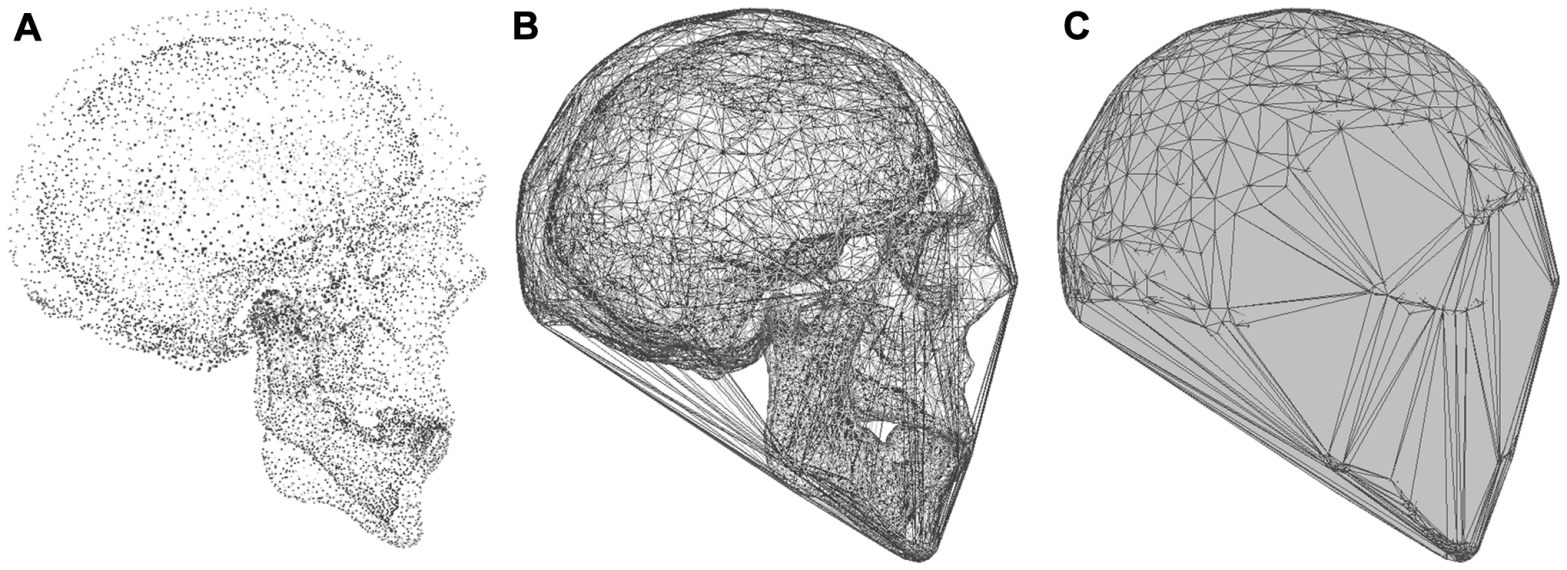
Convex hulling applied to a human skull. A, point cloud representing both the inner and outer surface contours of the skull; B, illustrates fit of the convex hull around the maximum extent of the skull; C, convex manifold (water-tight) polytope fitted by the hulling operation.

When applied to the problem of fossil mass estimation, the convex hulling process is used to calculate a minimum body volume from vertebrate skeletons. Digital models of the skeleton can be acquired using imaging techniques such as light radar (LiDAR), computed tomography (CT) or photogrammetry [24]. Whole skeletons are then segmented into functional units (i.e. trunk, thigh, skull etc.) and converted to point clouds (Figure 2A.). Each point cloud consists of a large dataset of points or vertices (typically ranging from 10^3^–10^6^ depending upon the functional unit in question) representing the surface of the skeletal element that are saved as *x, y,* and *z* coordinates. The convex hulling operation then works to fit the smallest convex polytope around that set of points, resulting in a tight-fitting hull around the skeleton and a minimum value for the convex wrapping volume (*vol*_CH_) (Figures 2B–C).

Rather than apply this technique directly to fossil skeletons, previous authors have used the convex hull method to derive calibration curves from modern species for mass prediction. Sellers et al. [18] calculated *vol*_CH_ in a range of quadrupedal mammals and multiply this value by an average density of 893 kg/m^−3^ to generate a minimum convex hull mass. This mass was then regressed against literature estimates for live body mass to produce a predictive equation. In contrast, Brassey et al. [8] directly regressed *vol*_CH_ against literature mass estimates when deriving a ratite-specific calibration curve in order to avoid uncertainty associated with assigning a particular density. There is however an implicit assumption that the predictive model is being applied to a fossil species closely related to (and hence likely to possess similar body density to) the modern calibration dataset. In this instance, a ratite-specific curve was applied to fossil moa.

Mass estimation techniques previously applied to hominid remains have been classified into two groups; ‘mechanical’ methods which rely upon a functional relationship between weight-bearing postcranial elements and mass, and ‘morphometric’ methods which directly reconstruct mass from preserved features such as bi-iliac breadth [25]. To the authors’ knowledge, whole-body volumetric mass estimation techniques have not previously been applied to hominids, or primates more generally, perhaps because of the often-fragmentary nature of the primate fossil record. However the hominin skeletons of AL 288-1 (*Australopithecus afarensis*) [26] and KNM-WT 15000 (*Homo erectus*) [27] are exceptional for early hominids in possessing a considerable proportion of limb bone and ribcage material, and a volumetric reconstruction may be feasible in these cases. Similarly, Miocene apes such as the African genus *Proconsul* [28] and new Spanish specimens including *Pierolapithecus* [29] are also known from reasonably complete skeletons, and there are also strepsirrhine examples such as *Darwinius* [30] and the giant lemurs of Madagascar [31].

In the case of linear predictive equations derived from limb bones (i.e. the ‘mechanical’ methods above), there are good biomechanical reasons why weight-bearing postcranial elements should be highly correlated with mass [32]. We know that convex hull calibration curves derived for modern species of birds and quadrupedal mammals are characterised by extremely high correlation coefficients (*r* ^2^ of 0.97 and 0.98 respectively). However before we apply this technique any further, it is prudent to likewise consider the biomechanical reasons why minimum body volume is informative with regards to body mass. Specifically, when *vol*_CH_ scaling departs from isometry in a group of vertebrates, how might this be interpreted?

The aims of this study are therefore:

- To derive a primate-specific convex hull calibration curve to complement those already existing for non-primate mammals and birds
- To compare *vol*_CH_ allometry between modern vertebrate groups
- To interpret the scaling behaviour of *vol*_CH_ in the context of interspecific variations in body density, composition and body plan.

## Methods

All skeletal material included in this study was accessed with the permission of the relevant museum or institution (University Museum of Zoology Cambridge, UMZC; Oxford University Museum of Natural History, OUMNH; Kyoto University Primate Research Institute, KUPRI; The National Library of Medicine, NLM) and reside within their permanent collections. A list of specimens is included in Table 1 and the convex hulling method has been described in detail elsewhere [8], [18]. Briefly, mounted skeletons of ratites (UMZC) and non-primate mammals (OUMNH) were scanned using a Z+F Imager 5010 and 5006i LiDAR respectively. The museum galleries containing the specimens were scanned several times from various angles to ensure adequate coverage of the skeletons. Registration and aligning of the LiDAR scans was carried out in Z+F LaserControl and individual skeletons were isolated and exported to Geomagic Studio v.12 (Geomagic, USA) as point clouds. CT scans of primate carcasses sourced from KUPRI, the human male sourced from the Visible Human Project (http://www.nlm.nih.gov/research/visible), plus additional CTs of two primates and two neognath birds from the University of Manchester were imported as DICOM files into OsiriX [34]. CT slice thickness ranged between 1–2.7 mm, with pixel spacings of 0.38–0.98 mm/pixel. The surface of each skeleton was rendered in 3D and exported to Geomagic Studio.

**Table 1:** Convex hull specimen list and sources of body mass.

species	accession no.	sex	volume (m^3^)	*M*_b_ (kg)	*M*_b_ source
*Struthio camelus*	UMZC374	–	7.17×10^−2^	60.7	[8]
*Casuarius casuarius*	UMZC371.D	–	1.72×10^−2^	27.0	[8]
*Dromaius novaehollandiae*	UMZC363	–	2.14×10^−2^	20.06	[8]
*Rhea americana*	UMZC378.gg	–	1.77×10^−2^	16.3	[8]
*Rhea pennata*	UMZC378ki	–	1.59×10^−2^	14.9	[8]
*Apteryx australis*	UMZC378.A	–	1.10×10^−3^	2.96	[8]
*Apteryx australis lawryi*	UMZC378.SS	F	1.40×10^−3^	2.41	[8]
*Branta leucopsis*	–	–	1.10×10^−3^	1.69	[*]
*Numida meleagris*	–	F	1.00×10^−−3^	1.40	[*]
*Bison bison*	OUMNH17430	M	4.73×10^−1^	558.5	[16]
*Bos taurus*	OUMNH17432	–	2.19×10^−1^	323.7	[16]
*Camelus dromedaries*	OUMNH17427	–	3.21×10^−1^	427.0	[16]
*Cervus elaphus*	OUMNH17431	M	8.40×10^−2^	89.5	[16]
*Dicerorhinus sumatrensis*	OUMNH4139	–	3.61×10^−1^	470.3	[16]
*Elephas maximus*	OUMNH10686	M	2.09×10^0^	2352.0	[16]
*Equus caballus*	OUMNH17428	–	3.70×10^−1^	517.5	[16]
*Giraffa camelopardalis*	OUMNH19507	–	4.35×10^−1^	638.2	[16]
*Loxodonta africana*	OUMNH4004	–	2.75×10^0^	2734.9	[16]
*Megaloceros giganteus*	OUMNH17433	–	3.01×10^−1^	435.6	[16]
*Rangifer tarandus*	OUMNH17529	–	7.57×10^−2^	95.8	[16]
*Sus scrofa*	OUMNH17426	–	7.79×10^−2^	107.4	[16]
*Tapirus indicus*	OUMNH17425	–	1.70×10^−1^	295.3	[16]
*Ursus maritimus*	OUMNH17459	–	1.11×10^−1^	206.1	[16]
*Chlorocebus aethiops*	KUPRI28	M	3.70×10^−3^	3.78	[*]
*Macaca fuscata*	KUPRI375	F	5.10×10^−3^	6.60	[*]
*Saimiri sciureus*	KUPRI290	F	6.00×10^−4^	0.759	[*]
*Hylobates agilis*	KUPRI277	M	5.40×10^−3^	6.75	[*]
*Hylobates lar*	KUPRI182	F	6.60×10^−3^	6.65	[33]
*Gorilla gorilla*	KUPRI298-317	M	9.57×10^−2^	176.0	[*]
*Pan troglodytes*	–	M	4.18×10^−2^	50.9	[33]
*Pongo pygmaeus*	–	F	3.25×10^−2^	45.0	[*]
*Homo sapiens*	NLM	M	4.91×10^−2^	68.9	[*]

UMZC, University Museum of Zoology, Cambridge; OUMNH, Oxford Museum of Natural History; KUPRI, Kyoto University Primate Research Institute; NLM, National Library of Medicine. Sources of body mass (*M*_b_); [*] carcass weight; [33] estimated using predictive equation for Hominoid body mass based upon radial head surface area (mm^2^) derived from CT images.

**Figure 3:**
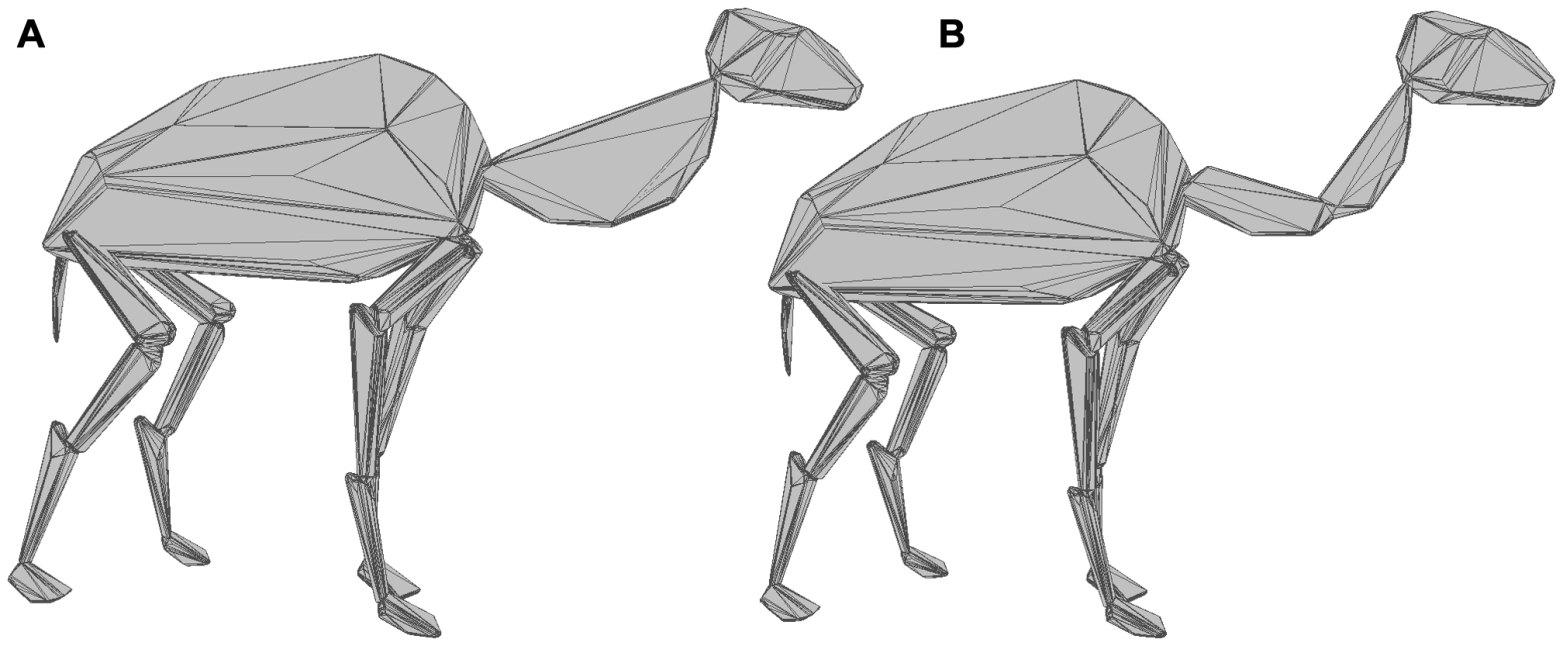
Effect of subdividing neck of *C*. *dromedaries* on convex hull volume. A, illustrates extent of neck convex hull without subdivision due to curvature of cervical series; B, tighter fit of convex hulls when divided into two parts.

Individual skeletons were subdivided into functional units. In the mammals, the body was divided into the trunk (including the pelvis, ribs, sternum and scapula), thigh (femur), shank (tibia), forearm (radius and ulna), upper arm (humerus), neck and skull. In the case of ungulates, the metatarsals and metacarpals were considered as separate segments from the phalanges. For all other mammals, the tarsals and phalanges were combined into one functional unit for hulling. The long necks of the giraffe (*Giraffa camelopardalis*) and camel (*Camelus dromedaries*) were segmented into two parts to ensure a tight-fitting hull around their length (Figure 3). The long tails of the grivet (*Chlorocebus aethiops*), squirrel monkey (*Saimiri sciureus*) and Japanese macaque (*Macaca fuscata*) were divided into multiple segments for the same reason. The antlers of the cervids were not included. The skeleton of the birds was subdivided into the trunk (pelvis, ribs, scapula and sternum plus keel and clavicle in the neognaths), thigh (femur), shank (tibiotarsus), tarsometatarsus, proximal wing (humerus), distal wing (radius and ulna), hand (metacarpals and phalanges), feet (phalanges), neck and skull. As in the long-necked mammals, bird necks were subdivided to ensure a tight fit.

The gorilla (*Gorilla gorilla*) was CT scanned as disarticulated body parts and some digital rearticulation of the skeleton was necessary prior to convex hulling. The lateral margins of both iliac crests had not been included in the CT and required restoration. The scapulae had been disassociated from the ribcage and were repositioned before convex hulling of the trunk. Furthermore the skull associated with the male gorilla carcass (KUPRI298-317) was not available, and the skull of a different male gorilla was scaled up geometrically based on limb length in its place. Both the lesser and greater apes in the sample were CT scanned lying in the supine position. In contrast the non-hominoid primates were scanned lying on their side. As a result, the latter group displayed considerable curvature of the spine dorsoventrally. This was corrected by straightening the spine in 3DsMax (Autodesk, USA) in order to ensure all primate trunks were of a comparable shape before hulling. CT data are available from http://www.pri.kyoto-u.ac.jp/and http://www.nlm.nih.gov/research/visible/, and convex hulls are available for download from http://www.animalsimulation.org.

Once subdivided, body segments were saved as.obj files. The convex hulling process was carried out in MATLAB (MathWorks, USA) using the ‘convhulln’ function. Convhulln implements the Quickhull (‘qhull’) algorithm for computing the convex hull [35]. Total *vol*_CH_ of a skeleton was calculated as the sum total of segment volumes. Body mass (*M*_b_, kg) was regressed against *vol*_CH_ (m^3^) for three groups (non-primate mammals, primates and birds) in MATLAB (see Table 1 for *M*_b_ sources). Slopes were fitted by means of ordinary least squares (OLS) regressions, as Type-I models are recommended when regressions will be used in a predictive capacity [6]. Slopes were compared in a one-way analysis of covariance (ANCOVA) using the ‘multcomp’ function in MATLAB, with subsequent pair-wise post hoc Tukey HSD test. Apparent density of the convex hulled skeleton (ρ_CH_, kg/m^−3^) was also calculated as *vol*_CH_/*M*_b_.

**Figure 4:**
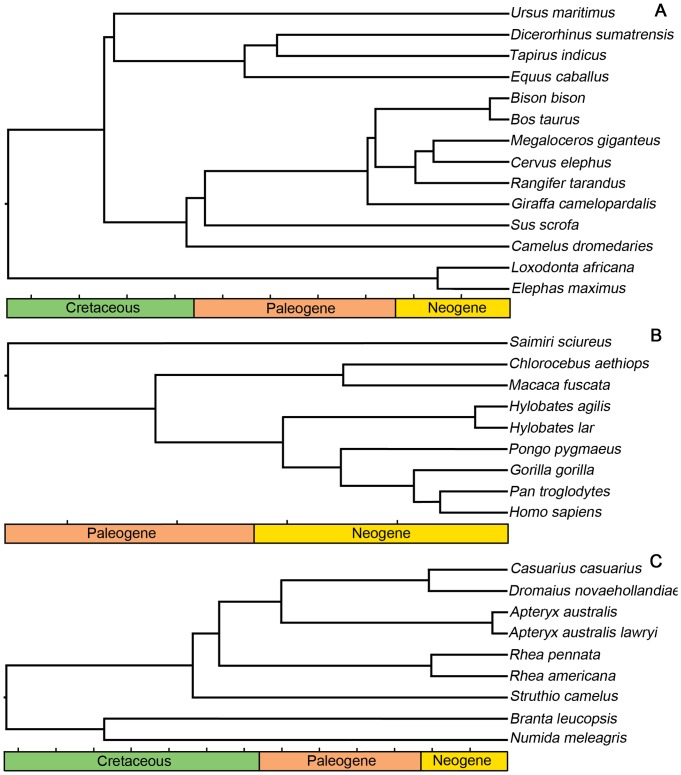
Consensus trees used in phylogenetic analysis. Tick marks represent increments of 10 million years. A, non-primate mammal tree topology and branch lengths derived from [37]–[44]; B, primate tree derived from [45]; C, bird tree derived from [46]–[48].

In order to account for evolutionary relationships, phylogenetically based regression models were also applied. This methodology is described in detail elsewhere [36]. Composite phylogenies were constructed in Mesquite ver. 2.75 (http://mesquiteproject.org) using tree topologies and branch lengths derived from previous publications (Figure 4A–C). The MATLAB program ‘Regressionv2.m’ [49] was used to implement multiple phylogenetic regressions. Phylogenetic generalised least squares (PGLS) assumes residuals are correlated due to shared ancestry and can be described by a Brownian motion model of evolution. Alternatively the Ornstein-Uhlenbeck (OU) evolutionary process models stabilising selection around an optimum [49]. The goodness-of-fit of the regression models is compared using uncorrected Akaike Information Criterion (AIC), in which smaller values imply a better fit. Models with an AIC value of <2 units greater than the minimum value are also said to have considerable support [50]. The optimal Ornstein-Uhlenbeck transformation parameter (*d*) was also estimated, where *d = *1 suggests the PGLS models fits the data better and *d* = 0 suggests a better fit for the OLS model.

## Results

**Figure 5:**
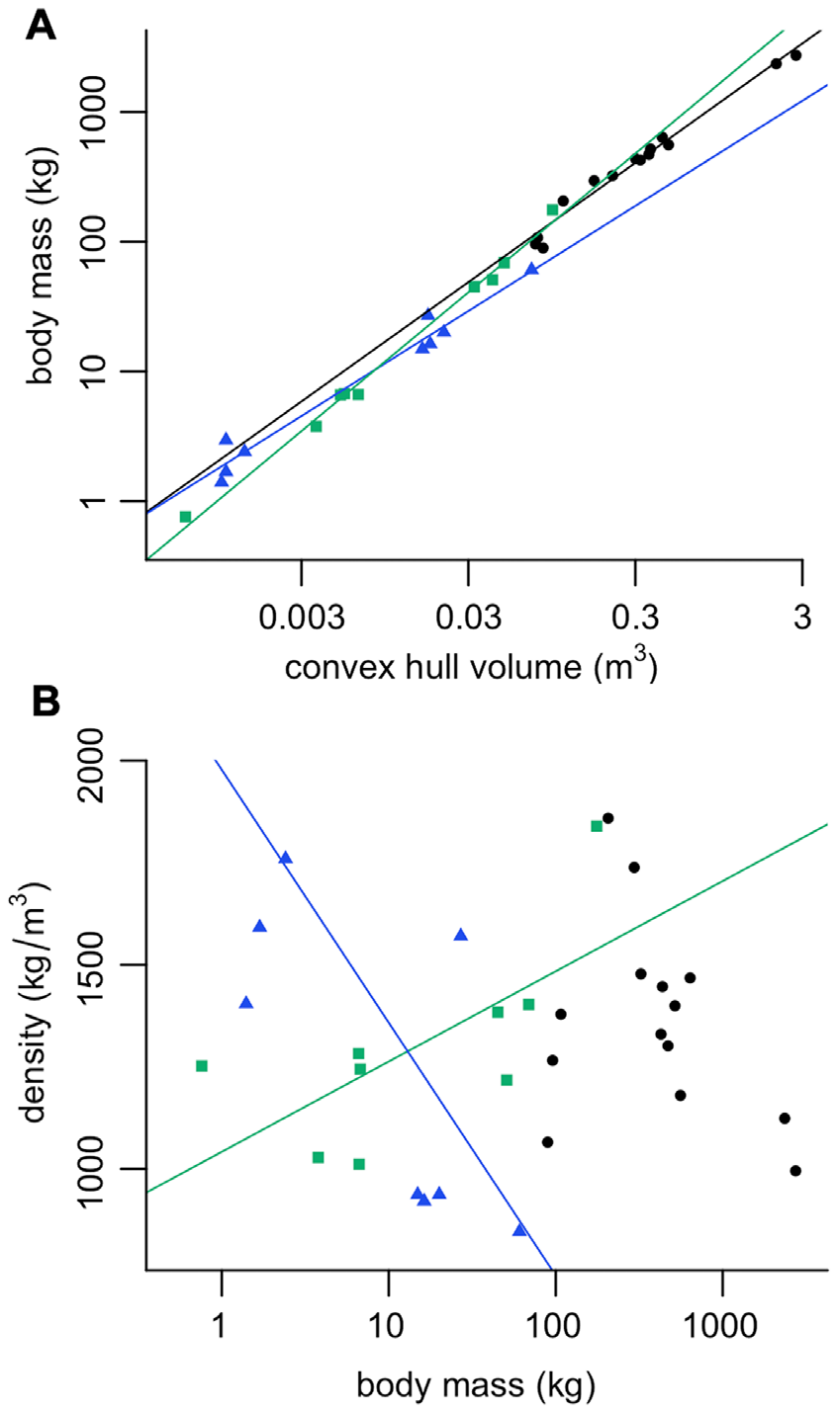
OLS regression results. A, Body mass (kg) against convex hull volume (m^3^). For slope equations, see Table 2 (labelled in bold). B, apparent density of convex hull (kg/m^3^) against body mass (kg). Density did not scale with body mass in non-primate mammals. Density increases with body mass in primates (*a* = 1042, *b* = 221, *r* ^2^ = 0.46, p = 0.025) yet decreases with body mass in birds (*a* = 1977, *b* = −619, *r* ^2^ = 0.36, p<0.05). Black circles, non-primate mammals; green squares, primates; blue triangles, birds.

Total *vol*_CH_ estimated for the skeletons are given in Table 1. The results of the OLS and phylogenetically corrected regressions of *M*_b_ against *vol*_CH_ are given in Table 2, and for OLS are plotted in Figure 5. Prior to log_10_ transformation the datasets did not meet the requirements for normality (Shapiro-Wilks test) and homoscedasticity (Breusch-Pagan test). Model results are therefore only reported for log_10_ transformed data. For all the groups considered here, the phylogenetically uncorrected OLS regression model provides a better fit to the data as indicated by lower AIC values for OLS models compared to PGLS and OU models (Table 2). This is further supported by *d* values of ≤0.011, again suggesting a better fit to the data in the OLS models than PGLS. The need for phylogenetic correction in this instance therefore remains equivocal, and for the sake of comparisons between our sample groups we only discuss log_10_ transformed OLS models further. However the potential for phylogenetic biasing, particularly of the primate slope, is considered further in the discussion.

**Table 2:** Ordinary least squares (OLS) and phylogenetically based regression (PGLS, phylogenetic generalised least squares; OU, Ornstein-Uhlenbeck process) of body mass (kg) against convex hull volume (m^3^).

	Fit	Type	*a*	*a* ±95%	*b*	*b* ±95%	*r* ^2^	AIC	*d*	MSE	%PE	PE ±95%
Mammals												
	**Log_10_**	**OLS**	**3.09**	**3.04–3.14**	**0.92**	**0.85–1.00**	**0.98**	**−31.2**	**–**	**0.005**	**11.6**	**6.96–17.1**
		PGLS	3.06	2.88–3.21	0.88	0.77–0.97	0.96	−16.9	–	–	–	
		OU	3.09	3.03–3.13	0.92	0.86–1.00	0.98	−29.2	<0.001	–	–	
Primates												
	**Log_10_**	**OLS**	**3.24**	**3.12–3.35**	**1.07**	**1.01–1.12**	**0.99**	**−19.7**	**–**	**0.004**	**10.8**	**3.97–17.6**
		PGLS	3.34	3.00–3.74	1.10	0.96–1.24	0.96	−11.8	–	–	–	
		OU	3.24	3.10–3.35	1.07	1.01–1.12	0.99	−17.7	0.011	–	–	
Birds												
	**Log_10_**	**OLS**	**2.70**	**2.50–2.93**	**0.81**	**0.71–0.90**	**0.97**	**−9.98**	**–**	**0.013**	**19.7**	**5.81–33.5**
		PGLS	2.43	1.71–3.05	0.71	0.44–0.97	0.77	2.09	–	–	–	
		OU	2.71	2.47–2.89	0.82	0.71–0.89	0.97	−7.98	<0.001	–	–	

AIC, Uncorrected Akaike Information Criterion; *d*, restricted maximum likelihood estimate of the Ornstein-Uhlenbeck transformation parameter; ±95%, 95% confidence intervals of the intercept and slope; MSE, mean square error of the regression; %PE, mean percentage prediction error of the regression; PE ±95%, 95% confidence interval of mean PE. Log_10_ transformed OLS regressions discussed throughout the text and are highlighted in bold.

Geometric similarity would predict *M*_b_ to scale to *vol*_CH_ with a slope of 1. Non-primate mammals do not scale *M*_b_ to *vol*_CH_ significantly differently from isometry (*b* = 0.92, 95%CI = 0.85–1.00, *p* = 0.08). In contrast, primates scale *M*_b_ to *vol*_CH_ significantly faster than isometry (*b* = 1.07, 95%CI = 1.01–1.12, *p* = 0.05) whilst birds scale *M*_b_ slower than predicted by geometry similarity (*b* = 0.81, 95%CI = 0.70–0.91, *p* = 0.011). Comparing all three models in a one-way analysis of covariance (ANCOVA) finds a significant difference between slopes (*F*_(2,26)_ = 7.18, *p*<0.003). A post-hoc Tukey test confirms birds scale *M*_b_ to *vol*_CH_ significantly slower than primates (*p*<0.05). No other pairwise comparison is significant however. Mean apparent density (*vol*_CH_/*M*_b_) was not significantly different between groups (*F* = 0.23, *p* = 0.80). Mean apparent density did not scale with *M*_b_ in non-primate mammals. However, mean apparent density was found to increase with *M*_b_ in primates (*r* ^2^ = 0.46, *p* = 0.025), and decrease with *M*_b_ in birds (*r* ^2^ = 0.36, *p*<0.05) (Figure 5B).

Due to the considerable amount of reconstruction work necessary on the gorilla skeleton prior to convex hulling and the resulting uncertainties in the placement of the scapulae and reconstruction of the ilium, the effect of excluding the gorilla individual from the sample was investigated. Removing the gorilla from the primate dataset did not significantly affect the slope of the regression line of *M*_b_ against *vol*_CH_ (*b* = 1.07 with gorilla, *b = *1.03 without gorilla, *p* = 0.46). However, primates scale *M*_b_ to *vol*_CH_ with isometry (*p>*0.34) and apparent density no longer scales with *M*_b_ in primates (*r* ^2^ = 0.21, *p*>0.12) when the gorilla is removed from the sample.

In this study, data were collected using two imaging techniques (CT and LiDAR). We investigated how the choice of imaging technique might impact upon our results by exploring the relationship between body size of the specimen, point cloud density and *vol*_CH_ of the trunk. This sensitivity analysis was conducted on the trunk segment rather than the whole body set, as the trunk comprises the vast majority of total *vol*_CH_ and any sampling effect demonstrable on the trunk will almost certainly be present in the whole body model. In the CT-scanned specimens, no relationship exists between *M*_b_ (used as a proxy for total body size) and the number of points comprising the trunk (*p*>0.05). This is because pixel size is manually adjusted for each individual during scanning in order to achieve the highest resolution possible.

In LiDAR-scanned skeletons, there is a significant correlation between *M*_b_ and number of points comprising the trunk (*p<*0.05 for LiDAR birds, *p*<0.01 for LiDAR non-primate mammals). As the LiDAR skeletons were isolated from one larger LiDAR point cloud of the surrounding museum gallery, larger individuals consist of a greater number of points than smaller individuals. The point clouds of trunk segments were randomly subsampled down in Geomagic Studio, such that all individuals comprised an equal number of points. In a paired Student’s t-test, no significant difference existed in *vol*_CH_ of the trunk between original and down-sampled point clouds in the UMZC ratites (*t = *1.97, *df = *8, *p*>0.05) and OUMNH non-primate mammals (*t* = 2.04, *df* = 13, *p*>0.05). Furthermore, the scaling exponents of *M*_b_ to trunk *vol*_CH_ were not significantly different between original and down-sampled point clouds in ratites (*p*>0.99) and non-primate mammals (*p*>0.96).

## Discussion

### Convex Hull Mass Estimation

*M*_b_ correlates extremely well with *vol*_CH_ in modern birds and mammals (*r* ^2^ = 0.97–0.99) and mean percentage prediction errors (%PE) of the models are encouragingly low (Table 2). Our values for mean %PE (11–20%, Table 2) compare favourably with bivariate predictive models recently derived from limb bones of mammals (25–71%PE, [2]) and volant birds (13–128%PE, [3]) comprising much larger datasets. The 95% confidence intervals on our mean %PE are similar to those of Campione & Evans [2], but are considerably wider than those of Field et al. [3]. The application of convex hulling to the problem of body mass estimation in fossil species is therefore justifiable when a large proportion of the skeleton is preserved. The authors have previously applied this mass estimation technique to fossil dinosaurs and birds, and here we present a primate-specific calibration curve of interest to those in the field of physical anthropology.

Primates are found to scale *M*_b_ to *vol_CH_* similarly to non-primate mammals (*p*>0.05). That primates are found to scale their skeletal dimensions similarly to other mammals is not without precedent. The scaling exponents of primate forelimb and hindlimb length to body mass overlap those of Carnivora, Rodentia and Scandentia [51], [52], with Marsupials the only order in this study to scale hindlimb length significantly differently from primates [51]. Similarly Polk et al. [53] found the confidence intervals of primate-specific regressions of hindlimb bone length and cross-sectional properties against mass to overlap considerably with those of Carnivora and Rodentia.

Therefore combining the non-primate mammal and primate datasets, a general mammal calibration curve is derived (*a* = 3.13, *b* = 1.011, *r* ^2^ = 0.993, *p*<0.001, mean square error (MSE) = 0.0052, mean percentage prediction error (%PE) = 12.0%). Given the log-transformed nature of the data, caution should be exercised if the equations presented here are to be subsequently applied to fossil skeletons in a mass prediction capacity. When back-transforming a linear model of the form:
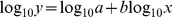
(1)into a power function of the form:
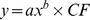
(2)a correction factor (CF) should be applied, which is calculated as:
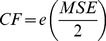
(3)where MSE is the mean square error of the regression [54]. Multiplying by the correction factor converts the geometric mean value of *y* calculated by taking the antilog of log(*y*) into an arithmetic mean value of *y*. Values of MSE for our regression models are provided in Table 2 for this purpose. However, given the extremely high correlation coefficients and low values for MSE charactering our models, multiplying by the correction factor will have very little effect on convex hull mass predictions.

Phylogenetic analyses have indicated some degree of biasing of the primate slope due to shared evolutionary history (Table 2). Our limited sample is dominated by hominoids (greater and lesser apes constitute two thirds of the primates included). The predictive equation derived here is still applicable to the field of early human evolution, for example, but should be cautiously applied to other primate groups that are not represented in our sample. Furthermore, the performance of PGLS regressions when predicting the body mass of species not included in the original regression remains unclear, and the application of both OLS and PGLS is recommended [55].

The factor limiting the application of this methodology to physical anthropology will be a lack of appropriate specimens. The relative paucity of associated postcranial hominin fossil material at present makes the widespread use of this calibration curve unlikely, and any such attempt would almost certainly require significant reconstruction. This highlights one of the potential concerns regarding the convex hull mass estimation technique. Whilst our methodology removes the need for authors to subjectively recreate soft tissue morphology by working on the skeleton alone, this acts to shift the burden of subjectivity onto those responsible for skeletal mounting of museum specimens. The flaring of the ribcage [16], positioning of the sternum [8], intervertebral spacing [16], [56] extent of cartilaginous epiphyses [57] and placement of the scapulae are known to effect mass estimates and biomechanical functionality of fossil reconstructions.

Recent efforts to remount fossil specimens, such as the Berlin *Brachiosaurus brancai* [58], according to our current understanding of their biology are commendable. However in most instances it is not feasible to physically remount skeletons due to time and financial constraints, alongside the potential for damage to the specimen. In this case, convex hulling provides a solution. The digital nature of our volumetric models allows skeletal components to be easily manipulated and whole skeletons may be digitally remounted. Sensitivity analyses on both the skeletal mount and soft tissue reconstructions are therefore entirely feasible, and should be a prerequisite for functional analyses.

### Scaling of Body Mass with vol_CH_

Non-primate mammals scale body mass isometrically with respect to *vol*_CH_, and apparent density is mass invariant (Figure 5B). However in modern primates and birds, *vol*_CH_ scales allometrically and apparent density therefore changes with body mass (Figure 5B). This may be interpreted in one of two ways:

- The allometric scaling of apparent density reflects a real trend in scaling of carcass density to body mass in birds and primates.
- Carcass density is actually mass-independent, yet convex hulling (and apparent density) is capturing a shift in the distribution of body tissue with size in these groups.

Convex hull volume is certainly an underestimate compared to fleshed-out body volume (*vol*_fl_) as it neglects any muscle, fat or integument that would have sat outside the contours defined by the maximum extent of the hard tissues. This is confirmed by the extremely high values for mean apparent density calculated here (primates = 1296 kg/m^3^, non-primate mammals = 1359 kg/m^3^, birds = 1418 kg/m^3^) compared to values for whole body density throughout the literature (see later discussion and Table S1)**.**

However, the degree to which *vol*_CH_ underestimates *vol*_fl_ depends on the distribution of such soft tissues, and this will vary both within and between skeletons. Within the hind limb for example, a greater mass of muscle is held proximally with the distal joints instead being controlled by long tendons [59]. Hence, *vol*_CH_ will be a smaller proportion of *vol*_fl_ around the thigh compared to the shank and feet. Likewise, interspecific variation in the amount of soft tissue held outside the convex hull envelope will cause variation in *vol*_CH_: *vol*_fl_, and apparent density values between species.

In light of this, it is unsurprising that non-primate mammals scale *vol*_CH_ isometrically and apparent density does not change with body mass. Our sample consists entirely of terrestrial species without specialist adaptations for climbing, swimming or digging. The bauplan (ground plan of the body segments) is therefore relatively well conserved throughout the sample (with the exception of the giraffe’s neck and camel’s hump). Apparent densities do vary considerably (Figure 5B) but do not scale to body mass.

When including the gorilla in the dataset, we find primates to scale *M*_b_ to *vol*_CH_ with positive allometry (*b* = 1.07, 95%*CI* = 1.01–1.12) and hence apparent density increases with mass. With the exception of humans, there are extremely sparse data in the literature regarding primate body density (Table S1) and without additional information on non-humans, a conclusion regarding the possible scaling of carcass density cannot be reached. Alternatively, apparent density may be scaling due to a size-related shift in the distribution of soft-tissue around the skeleton. Unpublished data has found terrestrial primates to be more muscular than arboreal species, regardless of their taxonomic affinity [60]. The largest members of our primate sample (*G. gorilla, Homo sapiens, Pan troglodytes*) are either entirely or primarily terrestrial. As such, we might expect the increase in apparent density in terrestrial apes to reflect increased muscle mass held outside the convex hull envelope, and therefore an increase in the *vol*_fl_: *vol*_CH_ ratio. However, the scaling exponent for primate *M*_b_ to *vol*_CH_ is barely above isometry, and when the gorilla is removed from the dataset due to concerns regarding the reconstruction of the disarticulated skeleton, there is no significant relationship between primate apparent density and body mass (*p*>0.05). This suggests our results are very sensitive to taxon sampling and more data regarding primate segment density and body composition, to compliment the wealth of existing data regarding segment mass and inertial properties, are sorely needed to resolve this uncertainty. Furthermore, two primates included in this study did not possess associated body mass data (*Hylobates lar, Pan troglodytes*). Despite being captive animals, literature values for mass were assigned to these specimens based upon regressions derived from wild-collected specimens (see Table 1). Primate individuals residing in zoos are known to be heavier, possess a higher body mass index (BMI) and percentage body fat composition than wild individuals [61] and our assigned values for body mass are therefore likely to be underestimates.

Despite the reputation of birds as being comparatively ‘light-weight’ [62], here we find the apparent densities of some avian individuals to be higher than those of modern mammals (Figure 5B). Due to the variety of methodologies employed to calculate carcass density and inconsistencies in the way in which density is reported in the literature, a statistical meta-analysis of previously published values is not possible. With the exception of diving birds however, a trend is visible in the literature whereby carcass density appears to be lower in birds than mammals (Table S1). This divergence between apparent convex hull density and carcass density may therefore be attributed to the convex hulling process itself.

Hypothesised adaptions or exaptations for weight saving in modern birds include possession of more hollow long bones ([63], although see [64]–[65]) and pneumatisation of the postcranial skeleton [66]. The convex hulling process does not account for the presence of air-filled cavities of a much lower density than soft tissue, resulting in inflated values for apparent density relative to carcass density. This is not a concern when applying the *M*_b_ ∝ *vol*_CH_ model in a predictive capacity, assuming the degree of pneumaticity also changes in a predictable way with mass. No explicit data exists regarding the scaling of air-sac volume in modern Aves, however a positive relationship has been identified between body mass and a ‘pneumaticity index’ (scoring the presence/absence of pneumaticity in 12 anatomical units) in 37 species of bird [67]. It is not clear how applicable these results are to the bird calibration curve presented here, given that flightless ratites are not included in their sample. Further work is needed to quantify and incorporate segment-specific variation in body density into avian convex hulls, particularly when the calibration curves are to be subsequently applied to pneumatic saurischian dinosaur and fossil bird skeletons.

**Figure 6:**
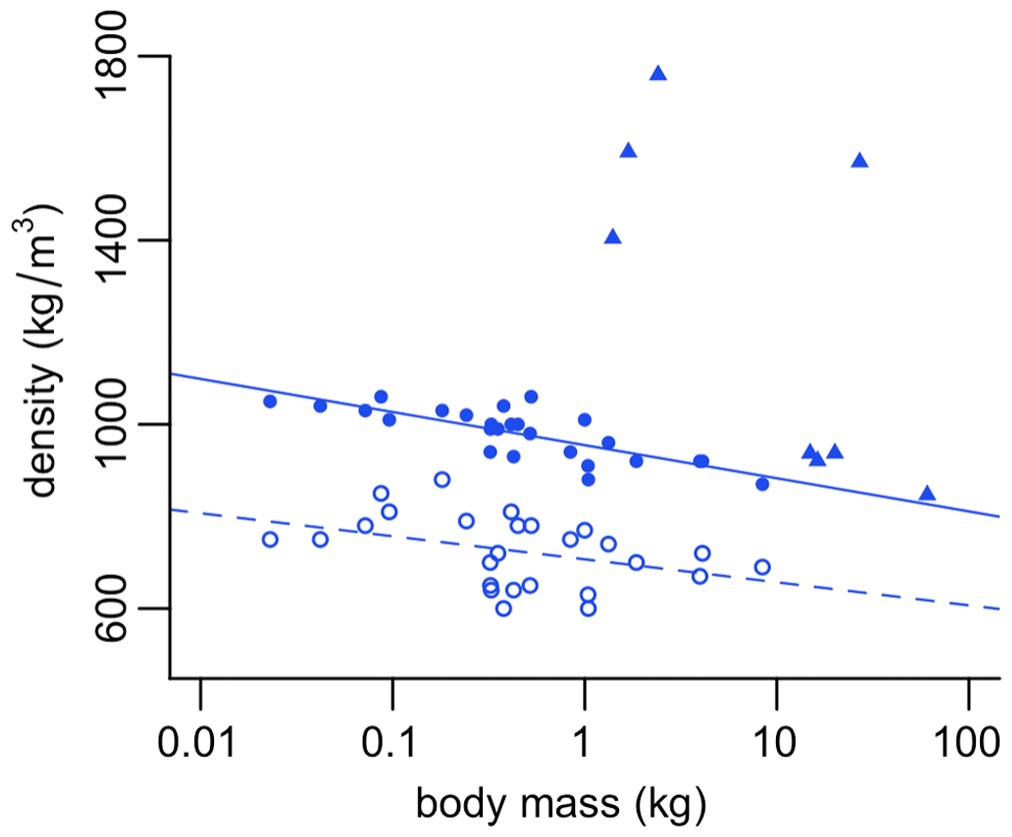
Scaling of density against mass in birds. Feathered carcass density (open circles) scales negatively against body mass (*a* = 707, *b* = −50.4, *r* ^2^ = 0.16, *p* = 0.04). Plucked carcass density (closed circles) scales negatively against body mass (*a* = 955. *b* = −72.4, *r* ^2^ = 0.61, *p*<0.001). Note the extremely weak correlation between feathered body density and mass, compared to the much stronger correlation between plucked carcass density and mass (see text for further discussion). Feathered and plucked carcass data from Budgey (2000). Convex hull density in birds also given for reference (closed triangles).

Here we find a significant negative relationship between apparent density and body mass within our bird sample (Figure 5B). Re-analysing previously published data [68], in which the feathered and plucked body densities of 26 species of neognath birds were estimated using fluid displacement, a negative relationship is also found (Figure 6). Interestingly plucked carcass density is found to have a much stronger correlation to body mass than feathered density (plucked *r* ^2^ = 0.61, *p*<0.001, feathered *r* ^2^ = 0.16, *p* = 0.04). Given feather mass appears to scale isometrically with high correlation coefficients in neognaths [69]–[70], this may be attributed to variability in the volume of air trapped beneath feathers and/or methodological difficulties associated with air escaping prior to submergence.

Superimposing our data points for apparent density onto those calculated by Budgey [68] (Figure 6), our values for large ratites (*Struthio camelus, Rhea americana, Rhea pennata, Dromaius novaehollandiae*) fall very close to those predicted by the plucked carcass model. An exception is the cassowary (*Casuarius casuarius*) which has previously been identified as an outlier in a ratite-specific convex hull calibration curve due to uncertainties in a literature-assigned body mass [8]. In contrast, the smaller ratites (*Apteryx australis, Apteryx australis lawryi*) and neognaths possess apparent densities greatly in excess of those predicted for plucked carcasses. This suggests that the volume of ‘missing’ soft tissue located outside the convex hull is greater in smaller birds.

The pectoral muscles constitute the largest organ in flying birds, comprising on average 17% of total body mass [71]. In contrast, pectoral muscle in flightless ratites is considerably reduced relative to volant species [72]–[73], with pectoralis mass accounting for 0.25% of total mass in kiwi [74]. However variation in pectoralis mass is unlikely to account for the observed trend in apparent densities, as the possession of large pectoralis muscles has an osteological correlate in the occurrence of a keeled sternum. The keel will act to increase *vol*_CH_ in neognaths by shifting the maximum extent of the convex hull ventrally, and thus account for the presence of an enlarged pectoralis musculature.

As a counterpoint, the reduction in pectoral musculature in ratites is accompanied by an increase in pelvic musculature relative to flighted birds. Values for hindlimb muscle mass as a percentage of total *M*_b_ for ostrich (*Struthio camelus*) range from 29% [75] to 34% [76], and values of 25% are reported for the emu (*Dromaius novaehollandiae*) [77]. In contrast, the lower extremities of flighted neognaths (including muscle and skeletal parts) account for 1–17% of body mass in a diverse sample of species [78]. The exclusion of hindlimb musculature by the convex hulling process cannot account for the observed trend in apparent densities however, as proportionally more muscle mass would be excluded from ratite convex hulls than flighted birds. This would results in an *increase* in apparent density in ratites relative to neognaths, the reverse of the trend observed in this study.

As previously discussed, feather mass is known to scale isometrically with body mass in neognath birds [69]–[70], averaging 6% of total *M*_b_. A review of literature-reported values for feather mass suggests kiwis also fall within this range (4.7–6.8% of total *M*_b_, [79]). However, large ratites (ostrich, emu and rhea) are found to posses considerably less plumage (1.5–1.9% of total *M*_b_, see Table S2) than neognaths. Thus feather mass may account for a small proportion of the observed ‘missing mass’ in neognaths and kiwi, but cannot adequately explain such a large disparity between our apparent density values and plucked density values of Budgey [68] as observed in small birds. It does however highlight the importance of choosing appropriate modern analogues when reconstructing mass in fossil species. Alexander’s [80] estimates of moa body mass, incorporating a value for feather mass of 5.6% of total *M*_b_ (his method (i)), are likely to be overestimates given the plumage values for large ratites presented here.

In addition to muscle and integument, fat deposits are also stored outside the convex hull of the skeleton. Reanalysing the data presented by Daan et al. [81] on 22 neognath species, the average percentage body fat is 7.8% and this does not scale with body mass (*p*>0.36). Caution must be exercised however, as this study includes long-distance migratory species (known to lay down extensive fat deposits prior to departure) without clarifying the season of data collection. Similar values for average percentage body fat are found for ostrich (5%, [82]) and rhea (7%, [83]), whilst emu body fat composition is exceptionally high due to selection for oil production (28% body fat, [84]).

Regardless of whether total fat mass conforms to isometry, the *distribution* of adipose tissue across the body is highly uneven both within- and between bird species and this is likely to be reflected in the ‘missing mass’ of the convex hulling process. Wirestam et al. [85] found fat accumulation did not follow a geometrical model in flying birds, with deposition occurring preferentially at the front and back of the body. Neognaths preferentially deposit fat subcutaneously across the abdomen area (from sternum to cloaca) and in the furcula depression [86]. In contrast, large ratites (ostrich, emu, and rhea) are said to possess ‘minimal’ abdominal subcutaneous fat deposits, with a thick layer of adipose tissue stored within the retroperitoneum [87]. Very little body composition data exists for the kiwi, except for an average fat mass of 300 g given for *Apteryx mantelli* [79]. Assuming an average body mass of 1930 g for males and 2360 g for females, this represents a percentage body fat of 13–16% which is stored subcutaneously [79]. High apparent density values calculated here for neognaths and kiwi may therefore reflect a shift in the distribution of body fat to anatomical positions located beyond the convex hull extent defined by the skeleton.

Initial convex hull studies employed museum-based LiDAR scanning as a means of generating a modern calibration dataset [8], [18]. LiDAR allows a large dataset (a gallery full of skeletons, for example) to be acquired within 2–3 hours. However skeletal mounts on display in public museums may be mounted incorrectly, and frequently have no body mass data associated with them. In this case body masses must be subsequently assigned using literature values. Here, for the first time, we have derived a primate convex hull calibration curve using CT scan data of whole carcasses. This approach avoids problems associated with skeletal mounting (intervertebral spacing, scapula placement etc. are all predefined by the soft tissue still present in the scan), and mass can be recorded directly from the carcass. As CT is becoming cheaper and easier to access, this is a promising area for further research. Furthermore, incorporating magnetic resonance imaging (MRI) data has the potential to illuminate interspecific variation in muscle and fat mass distribution around the skeleton that has been discussed in some detail here.

## Conclusions

In summary, we have demonstrated that minimal convex hull volume (*vol*_CH_) is an extremely good predictor of body mass in modern groups of non-primate mammals, primates and birds. Our models are characterised by low values for mean percentage prediction error (%PE) equivalent to those recently reported for bivariate regressions of limb bone dimensions [2], [3] but with the added advantage of not relying upon single skeletal elements. We have highlighted the potential for the convex hulling method to be applied either solely for the purpose of estimating body mass in fossil species, or as a precursor to a functional biomechanical analysis for which body mass is a required input.

We have found, as expected, that the apparent densities of the convex hull objects calculated here are significant overestimates compared to published values of carcass density. This is due to the exclusion of ‘missing’ soft tissue held outside the contours of the skeleton from our calculations of volume. We have postulated on the possible sources of this missing soft tissue including muscle, integument and body fat, and present data collated from the literature regarding animal body density and composition. We believe this will be of interest to those working in the field of fossil reconstruction, particularly on saurischian dinosaurs and fossil birds.

The convex hulling method presented here sidesteps the requirement for soft-tissue reconstruction prior to mass estimation, and provides a straightforward means to conduct sensitivity analyses of the skeletal mount. However, user subjectivity is not entirely eliminated as decisions must still be made regarding the division of the skeleton into ‘functional’ units prior to convex hulling. The subdivision of the neck, tail and tarsal/phalanges is necessary to ensure a tight-fitting hull, yet requires some degree of user input. In studies focused upon a specific group with a shared body plan (such as ratites, [8]), this process is unlikely to effect the outcome of the calibration curve. However in studies incorporating species of differing bauplans, the way in which the skeleton is segmented up may impact upon the ultimate result. The methodology for subdividing the skeleton must therefore be explicitly stated, and further work is needed to quantify the effect of segmentation upon mass prediction curves.

## Supporting Information

Table S1 **Literature values for bird and mammal body density.** The value for human body density was chosen from a recent study of healthy non-athletes [1]. An extensive literature exists on human body density values as a means of assessing body fat composition, but is beyond the scope of the present study. Caution should be exercised when interpreting the body density of domesticated farm animals in particular due to artificial selection for fat deposition. Furthermore, the studies listed below differ in both their methodology for estimating density (fluid displacement, volumetric models, kinematics), and the condition of the carcass (articulated vs. disarticulated, feathered vs. plucked, complete vs. eviscerated).
(DOC)

Table S2 **Literature values for percentage contribution of feathers to total body mass in birds.** Refer to the original source for sample sizes and further details of the methodology. Protocols differ in terms of weighing total feather mass (contour feathers *plus* down feathers) vs. contour feathers only; whether feathers are artificially dried prior to weighing; whether male and female plumage is considered separately or grouped.
(DOC)
