# Supplementary material for: Scaling of Convex Hull Volume to Body Mass in Modern Primates, Non-Primate Mammals and Birds
Source: PLoS One. 2014 Mar 11;9(3):e91691. doi: 10.1371/journal.pone.0091691 (PMC3950251; doi:10.1371/journal.pone.0091691)
Supplement: Table S1 — Literature values for bird and mammal body density. The value for human body density was chosen from a recent study of healthy non-athletes [1]. An extensive literature exists on human body density values as a means of assessing body fat composition, but is beyond the scope of the present study. Caution should be exercised when interpreting the body density of domesticated farm animals in particular due to artificial selection for fat deposition. Furthermore, the studies listed below differ in both their methodology for estimating density (fluid displacement, volumetric models, kinematics), and the condition of the carcass (articulated vs. disarticulated, feathered vs. plucked, complete vs. eviscerated). (DOC) [file pone.0091691.s001.doc]

**Table S1. Literature values for bird and mammal body density.** The value for human body density was chosen from a recent study of healthy non-athletes [1]. An extensive literature exists on human body density values as a means of assessing body fat composition, but is beyond the scope of the present study. Caution should be exercised when interpreting the body density of domesticated farm animals in particular due to artificial selection for fat deposition. Furthermore, the studies listed below differ in both their methodology for estimating density (fluid displacement, volumetric models, kinematics), and the condition of the carcass (articulated vs. disarticulated, feathered vs. plucked, complete vs. eviscerated).

| Species | Density | Source | Notes |
| --- | --- | --- | --- |
| Mammals (ex. primates) |  |  |  |
| *Equus ferus caballus* | 893kg/m^3^ | [2] | Weighted mean of segment densities (n=1) |
| *Bos primigenius* | 1056kg/m^3^ | [3] | Mean density of right half of beef steer carcass (n=48) |
| *Ovis aries* | 912kg/m^3^ | [3] | Mean carcass density (including fat) (7 studies) |
| *Sus scrofa* | 943kg/m^3^ | [3] | Mean carcass density (including fat) (6 studies) |
| *Mesocricetus auratus* | 1049kg/m^3^ | [4] | Mean carcass density of male hamsters (n=34) |
| *Phoca sibirica* | 1140-1220kg/m^3^ | [5] | Estimated from terminal speed of Baikal seals (n=4) |
| *Zalophus californianus* | 841-1275kg/m^3^ | [6] | California seal pups, mean 17 days old (n=354) |
| Primates |  |  |  |
| *Pan troglodytes* | 1037-1110kg/m^3^ | [7] | Segment densities *excluding trunk* (n=1) |
| *Pongo pygmaeus* | 1030-1116kg/m^3^ | [7] | Segment densities *excluding trunk* (n=1) |
| *Homo sapiens* | 1049kg/m^3^ | [1] | Male and female average of normal BMI (n=46) |
| Birds |  |  |  |
| *Anser anser* | 937kg/m^3^ | [8] | Plucked carcass (n=1) |
| Duck | 900kg/m^3^ | [9] | (n=1) |
| Flying birds | 730kg/m^3^ | [10] | Unnamed species, plucked with inflated air-sacs (n=12) |
| *Coturnix japonica* | 1053-1069kg/m^3^ | [11] | Plucked and eviscerated Japanese quail (n=80) |
| *Struthio camelus* | 888 kg/m^3^ | [12] | Plucked trunk of ostrich (n=1) |
| Laridae (gulls) | 940kg/m^3^ | [13] | Whole feathered carcasses with air-sacs (n=5) |
| Anatidae (dabbling ducks) | 980kg/m^3^ | [13] | Whole feathered carcasses with air-sacs (n=6) |
| Anatidae (diving ducks) | 990kg/m^3^ | [13] | Whole feathered carcasses with air-sacs (n=3) |
| Procellariidae (petrels) | 990kg/m^3^ | [13] | Whole feathered carcasses with air-sacs (n=10) |
| Alcidae (auks) | 1000kg/m^3^ | [13] | Whole feathered carcasses with air-sacs (n=2) |
| Gaviidae (divers) | 1060kg/m^3^ | [13] | Whole feathered carcasses with air-sacs (n=1) |
| Spheniscidae (penguins) | 1020kg/m^3^ | [13] | Whole feathered carcasses with air-sacs (n=2) |
| Phalacrocoricidae (cormorants) | 1030kg/m^3^ | [13] | Whole feathered carcasses with air-sacs (n=1) |
| *Gallus gallus* | 894kg/m^3^ | [14] | Estimated from CT volumetric model (n=1) |
| *Gallus gallus domesticus* | 953kg/m^3^ | [14] | Estimated from CT volumetric model (n=1) |
| *Gallus gallus domesticus* | 918kg/m^3^ | [15] | Whole feathered carcasses (n=1) |
| *Quiscalus quiscula* | 809kg/m^3^ | [15] | Whole feathered carcasses (n=1) |
| *Sturnus vulgaris* | 776kg/m^3^ | [15] | Whole feathered carcasses (n=1) |
| *Passer domesticus* | 751kg/m^3^ | [15] | Whole feathered carcasses (n=1) |
| *Molothrus ater* | 750kg/m^3^ | [15] | Whole feathered carcasses (n=1) |
| *Anas platyrhynchos* | 739kg/m^3^ | [15] | Whole feathered carcasses (n=1) |
| *Cathartes aura* | 700kg/m^3^ | [15] | Whole feathered carcasses (n=1) |
| *Leucophaeus atricilla* | 700kg/m^3^ | [15] | Whole feathered carcasses (n=1) |
| *Branta leucopsis* | 669kg/m^3^ | [15] | Whole feathered carcasses (n=1) |
| *Columba livia* | 648kg/m^3^ | [15] | Whole feathered carcasses (n=1) |
| *Larus delawarensis* | 644kg/m^3^ | [15] | Whole feathered carcasses (n=1) |
| *Larus smithsonianus* | 602kg/m^3^ | [15] | Whole feathered carcasses (n=1) |

1. Shafer KJ, Siders WA, Johnson LK, Lukaski HC (2010) Body density estimates from upper-body skinfold thicknesses compared to air-displacement plethysmography. Clin Nutr 29: 249–254. doi:10.1016/j.clnu.2009.09.002.

2. Buchner HHF, Savelberg HHCM, Schamhardtt HC, Barneveld A (1997) Inertial properties of Dutch Warmblood horses. J Biomech 30: 653–658.

3. Garrett WN (1967) Experiences in the use of body density as an estimator of body composition in animals. Body composition in animals and man. Columbia: University of Missouri. pp. 170–185.

4. Kodama AM (1971) In vivo and in vitro and body water determinations of body fat in the hamster. J Appl Physiol 31: 218–222.

5. Watanabe Y, Baranov E a, Sato K, Naito Y, Miyazaki N (2006) Body density affects stroke patterns in Baikal seals. J Exp Biol 209: 3269–3280. doi:10.1242/jeb.02402.

6. Luque SP, Aurioles-Gamboa D (2002) Estimation of body volume and body density in California sea lion pups. J Mar Biol Assoc UK 82: 1019–1022. doi:10.1017/S0025315402006550.

7. Crompton RH, Li Y, Alexander RM, Wang W, Gunther MM (1996) Segment inertial properties of primates: new techniques for laboratory and field studies of locomotion. Am J Phys Anthropol 99: 547–570. doi:10.1002/(SICI)1096-8644(199604)99:4&lt;547::AID-AJPA3&gt;3.0.CO;2-R.

8. Alexander R (1983) Allometry of the leg bones of moas (Dinornithes) and other birds. J Zool 200: 215–231.

9. Welty JC, Baptista M (1988) The Life of Birds. 4th Editio. Stamford: Cengage Learning.

10. Hazlehurst GA, Rayner JM V (1992) Flight characteristics of Triassic and Jurassic Pterosauria: an appraisal based on wing shape. Paleobiology: 447–463.

11. Tserveni A, Yannakopoulos A (1988) Specific gravity, carcass fat and prediction of fatness in quail carcasses. J Agric Sci 111: 95–98. doi:10.1017/S0021859600082861.

12. Hutchinson JR, Ng-Thow-Hing V, Anderson FC (2007) A 3D interactive method for estimating body segmental parameters in animals: Application to the turning and running performance of *Tyrannosaurus rex.* J Theor Biol 246: 660–680.

13. Wilson RP, Hustler K, Ryan PG, Burger AE, Christian E, Url S (1992) Diving Birds in Cold Water : Do Archimedes and Boyle Determine Energetic Costs ? Am Nat 140: 179–200.

14. Allen V, Paxton H, Hutchinson JR (2009) Variation in Center of Mass Estimates for Extant Sauropsids and its Importance for Reconstructing Inertial Properties of Extinct Archosaurs. Anat Rec 292: 1442–1461.

15. Hamershock DM, Seamans TW, Bernhard GE (1993) Determination of body density for twelve bird species. Flight Dynamics Directorate, Wright Laboratory.
