# Supplementary material for: Scaling of Convex Hull Volume to Body Mass in Modern Primates, Non-Primate Mammals and Birds
Source: PLoS One. 2014 Mar 11;9(3):e91691. doi: 10.1371/journal.pone.0091691 (PMC3950251; doi:10.1371/journal.pone.0091691)
Supplement: Table S2 — Literature values for percentage contribution of feathers to total body mass in birds. Refer to the original source for sample sizes and further details of the methodology. Protocols differ in terms of weighing total feather mass (contour feathers plus down feathers) vs. contour feathers only; whether feathers are artificially dried prior to weighing; whether male and female plumage is considered separately or grouped. (DOC) [file pone.0091691.s002.doc]

Table S2. Literature values for percentage contribution of feathers to total body mass in birds. Refer to the original source for sample sizes and further details of the methodology. Protocols differ in terms of weighing total feather mass (contour feathers *plus* down feathers) vs. contour feathers only; whether feathers are artificially dried prior to weighing; whether male and female plumage is considered separately or grouped.

| Species | Common name | Feather (% *M*_b_) | Source | Notes |
| --- | --- | --- | --- | --- |
| **Neognaths** |  |  |  |  |
| **ACCIPITRIFORMES** |  |  |  |  |
| *Haliaeetus leucocephalus* | Bald eagle | 12% | [1] | Contour feathers |
| **FALCONIFORMES** |  |  |  |  |
| *Falco tinnunculus* | Common kestrel | 10.4% | [2] | Dry total feather mass |
| **GRUIFORMES** |  |  |  |  |
| *Gallinula chloropus sandvicensis* | Hawaiian moorhen | 3.5% | [3] | Total feather mass |
| *Fulica atra* | Eurasian coot | 7.1% | [2] | Dry total feather mass |
| **GALLIFORMES** |  |  |  |  |
| *Meleagris gallopavo* | Wild turkey | 5.6% | [4] | Total feather mass |
| *Gallus gallus domesticus* | Broiler chicken | 5.8% (F) 6.0% (M) | [5] | Total feather mass |
| *Coturnix coturnix* | Common quail | 4.7% | [2] | Dry total feather mass |
| **PSITTACIFORMES** |  |  |  |  |
| *Melopsittacus undulatus* | Budgerigar | 9.98% | [6] | Total feather mass |
| *Agapornis sp.* | Lovebird | 7.38% | [6] | Total feather mass |
| **CHARADRIIFORMES** |  |  |  |  |
| *Arenaria interpres* | Ruddy turnstone | 7.4% | [2] | Dry total feather mass |
| *Pluvialis apricarius* | Golden plover | 4.7% | [2] | Dry total feather mass |
| *Limosa lapponica* | Bar-tailed godwit | 4.5% | [2] | Dry total feather mass |
| *Larus ridibundus* | Black-headed gull | 10.1% | [2] | Dry total feather mass |
| *Haematopus ostralegus* | Eurasian oystercatcher | 8.8% | [2] | Dry total feather mass |
| *Larus argentatus* | European herring gull | 10.7% | [2] | Dry total feather mass |
| *Calidris maritima* | Purple sandpiper | 15.6% | [7] | Contour feather mass |
| **COLUMBIFORMES** |  |  |  |  |
| *Streptopelia decaocto* | Eurasian collared dove | 9.6% | [2] | Dry total feather mass |
| *Zenaida macroura* | Mourning dove | 7.7% | [8] | Contour feather mass |
| **ANSERIFORMES** |  |  |  |  |
| *Aythya fuligula* | Tufted duck | 4.8% | [2] | Dry total feather mass |
| *Anas platyrhynchos* | Mallard duck | 6.4% | [2] | Dry total feather mass |
| *Anas platyrhynchos* | Mallard duck | 6.0% | [9] | Total feather mass |
| *Branta bernicla* | Brant goose | 8.0% | [2] | Dry total feather mass |
| *Aix sponsa* | Wood duck | 5.3% | [9] | Total feather mass |
| *Anas strepera* | Gadwall | 6.5% | [9] | Total feather mass |
| *Anas americana* | American wigeon | 5.7% | [9] | Total feather mass |
| *Anas discors* | Blue-winged teal | 5.8% | [9] | Total feather mass |
| *Anas carolinensis* | Green-winged teal | 6.0% | [9] | Total feather mass |
| *Aythya americana* | Redhead | 5.3% | [9] | Total feather mass |
| *Aythya collaris* | Ring-necked duck | 5.1% | [9] | Total feather mass |
| *Aythya affinis* | Lesser scaup | 4.4% | [9] | Total feather mass |
| *Lophodytes cucullatus* | Hooded merganser | 5.1% | [9] | Total feather mass |
| **CAPRIMULGIFORMES** |  |  |  |  |
| *Chordeiles minor* | Eastern nighthawk | 8.2% | [8] | Contour feather mass |
| **APODIFORMES** |  |  |  |  |
| *Archilochus colubris* | Ruby-throated hummingbird | 7.1% | [8] | Contour feather mass |
| **PASSERIFORMES** |  |  |  |  |
| **Tyrannidae** |  |  |  |  |
| *Tyrannus tyrannus* | Eastern kingbird | 7.5% | [8] | Contour feather mass |
| *Myiarchus crinitus* | Northern crested flycatcher | 7.4% | [8] | Contour feather mass |
| *Empidonax virescens* | Acadian flycatcher | 8.8% | [8] | Contour feather mass |
| *Contopus virens* | Eastern wood pewee | 7.5% | [8] | Contour feather mass |
| **Hirundinidae** |  |  |  |  |
| *Stelgidopteryx sp.* | Rough-winged swallow | 5.8% | [8] | Contour feather mass |
| *Hirundo rustica* | Barn swallow | 8.0% | [8] | Contour feather mass |
| **Covidae** |  |  |  |  |
| *Cyanocitta cristata* | Northern blue jay | 7.0% | [8] | Contour feather mass |
| *Pica pica* | Eurasian magpie | 8.5% | [2] | Dry total feather mass |
| *Corvus monedula* | Western jackdaw | 9.1% | [2] | Dry total feather mass |
| *Corvus corone* | Carrion crow | 7.6% | [2] | Dry total feather mass |
| **Paridae** |  |  |  |  |
| *Poecile carolinensis* | Carolina chickadee | 6.9% | [8] | Contour feather mass |
| *Parus major* | Great tit | 9.7% | [2] | Dry total feather mass |
| *Poecile gambeli* | Mountain chickadee | 4.1% | [10] | Contour feather mass |
| *Baeolophus ridgwayi* | Juniper titmouse | 5.3% | [10] | Contour feather mass |
| **Certhiidae** |  |  |  |  |
| *Certhia americana* | Brown creeper | 7.1% | [8] | Contour feather mass |
| **Troglodytidae** |  |  |  |  |
| *Troglodytes aedon* | Eastern house wren | 5.3% | [8] | Contour feather mass |
| *Thryothorus ludovicianus* | Carolina wren | 3.3% | [8] | Contour feather mass |
| *Cistothorus palustris* | Long-billed marsh wren | 4.4% | [8] | Contour feather mass |
| **Mimidae** |  |  |  |  |
| *Mimus polyglottos* | Mockingbird | 7.0% | [8] | Contour feather mass |
| *Dumetella carolinensis* | Catbird | 6.5% | [8] | Contour feather mass |
| *Toxostoma rufum* | Brown thrasher | 4.6% | [8] | Contour feather mass |
| **Turdidae** |  |  |  |  |
| *Hylocichla mustelina* | Wood thrush | 5.3% | [8] | Contour feather mass |
| *Catharus guttatus* | Eastern hermit thrush | 7.4% | [8] | Contour feather mass |
| *Turdus merula* | Common blackbird | 9.3% | [2] | Dry total feather mass |
| **Regulidae** |  |  |  |  |
| *Regulus satrapa* | Eastern golden-crowned kinglet | 11.1% | [8] | Contour feather mass |
| *Regulus calendula* | Eastern ruby-crowned kinglet | 8.9% | [8] | Contour feather mass |
| **Laniidae** |  |  |  |  |
| *Lanius ludovicianus* | Migrant shrike | 6.1% | [8] | Contour feather mass |
| **Vireonidae** |  |  |  |  |
| *Vireo griseus* | White-eyed vireo | 5.2% | [8] | Contour feather mass |
| *Vireo flavifrons* | Yellow-throated vireo | 6.0% | [8] | Contour feather mass |
| *Vireo olivaceus* | Red-eyed vireo | 5.0% | [8] | Contour feather mass |
| **Parulidae** |  |  |  |  |
| *Mniotilta varia* | Black and white warbler | 4.4% | [8] | Contour feather mass |
| *Oreothlypis peregrina* | Tennessee warbler | 6.5% | [8] | Contour feather mass |
| *Setophaga pitiayumi* | Southern parula warbler | 5.2% | [8] | Contour feather mass |
| *Setophaga magnolia* | Magnolia warbler | 4.6% | [8] | Contour feather mass |
| *Setophaga caerulescens* | Black-throated blue warbler | 6.2% | [8] | Contour feather mass |
| *Setophaga virens* | Black-throated green warbler | 6.5% | [8] | Contour feather mass |
| *Setophaga fusca* | Blackburnian warbler | 4.5% | [8] | Contour feather mass |
| *Setophaga pensylvanica* | Chestnut-sided warbler | 5.8% | [8] | Contour feather mass |
| *Setophaga castanea* | Bay-breasted warbler | 5.0% | [8] | Contour feather mass |
| *Setophaga castanea* | Black-poll warbler | 6.8% | [8] | Contour feather mass |
| *Setophaga pinus* | Northern pine warbler | 7.9% | [8] | Contour feather mass |
| *Seiurus aurocapilla* | Oven-bird | 6.9% | [8] | Contour feather mass |
| *Parkesia motacilla* | Louisiana water-thrush | 5.1% | [8] | Contour feather mass |
| *Geothlypis formosa* | Kentucky warbler | 4.9% | [8] | Contour feather mass |
| *Oporornis agilis* | Connecticut warbler | 7.6% | [8] | Contour feather mass |
| *Geothlypsis trichas brachidactyla* | Northern yellowthroat | 6.0% | [8] | Contour feather mass |
| *Geothlypsis trichas trichas* | Maryland yellowthroat | 7.4% | [8] | Contour feather mass |
| *Icteria virens* | Yellow-breasted chat | 6.0% | [8] | Contour feather mass |
| *Cardellina canadensis* | Canada warbler | 8.5% | [8] | Contour feather mass |
| **Passeridae** |  |  |  |  |
| *Passer domesticus* | House sparrow | 5.3% | [8] | Contour feather mass |
| *Passer domesticus* | House sparrow | 8.6% | [2] | Dry total feather mass |
| **Icteridae** |  |  |  |  |
| *Agelaius phoeniceus* | Eastern red-wing | 5.2% | [8] | Contour feather mass |
| *Icterus spurius* | Orchard oriole | 6.3% | [8] | Contour feather mass |
| *Quiscalus quiscula* | Purple grackle | 7.1% | [8] | Contour feather mass |
| *Molothrus ater* | Eastern cowbird | 4.6% | [8] | Contour feather mass |
| **Cardinalidae** |  |  |  |  |
| *Piranga olivacea* | Scarlet tanager | 6.2% | [8] | Contour feather mass |
| *Pheucticus ludovicianus* | Rose-breasted grosbeak | 3.5% | [8] | Contour feather mass |
| *Passerina cyanea* | Indigo bunting | 5.6% | [8] | Contour feather mass |
| **Fringillidae** |  |  |  |  |
| *Carduelis tristis* | Eastern goldfinch | 6.0% | [8] | Contour feather mass |
| *Serinus canaria domestica* | Canary | 13.9% | [6] | Total feather mass |
| **Emberizidae** |  |  |  |  |
| *Pipilo erythrophthalmus* | Red-eyed towhee | 7.4% | [8] | Contour feather mass |
| *Passerculus sandwichensis* | Savannah sparrow | 7.3% | [8] | Contour feather mass |
| *Ammodramus savannarum* | Grasshopper sparrow | 5.5% | [8] | Contour feather mass |
| *Ammodramus henslowii* | Henslow’s sparrow | 6.2% | [8] | Contour feather mass |
| *Ammodramus sp.* | Sharp-tailed sparrow | 5.4% | [8] | Contour feather mass |
| *Ammodramus maritimus* | Northern seaside sparrow | 6.2% | [8] | Contour feather mass |
| *Pooecetes gramineus* | Vesper sparrow | 5.3% | [8] | Contour feather mass |
| *Spizella passerina* | Chipping sparrow | 5.6% | [8] | Contour feather mass |
| *Spizella pusilla* | Field sparrow | 7.2% | [8] | Contour feather mass |
| *Zonotrichia albicollis* | White-throated sparrow | 6.8% | [8] | Contour feather mass |
| *Melospiza melodia* | Song sparrow | 5.3% | [8] | Contour feather mass |
| *Junco hyemalis* | Dark-eyed junco | 3.4-4.1% | [11] | Dry total feather mass |
| **Estrildidae** |  |  |  |  |
| *Poephila guttata* | Zebra finch | 5.6% | [2] | Dry total feather mass |
| *Lonchura striata* | White-rumped munia | 3.8% | [2] | Dry total feather mass |
| **Muscicapidae** |  |  |  |  |
| *Erithacus rubecula* | European robin | 9.0% | [2] | Dry total feather mass |
| *Saxicola torquata rubicula* | European stonechat | 6.4% | [12] | Feather mass |
| *Saxicola torquata axillaris* | East African stonechat | 7.2% | [12] | Feather mass |
| **Maluridae** |  |  |  |  |
| *Malurus cyaneus* | Superb fairy-wren | 3.6% | [13] | Dry contour mass |
| **Palaeognaths** |  |  |  |  |
| *Apteryx sp.* | Kiwi | 4.7-6.8% | [14] | Total feather mass |
| *Struthio camelus* | Ostrich | 1.5% (F) & 1.7% (M) | [15] | Total body feathers |
| *Struthio camelus* | Ostrich | 1.9% | [16] | Feather mass |
| *Rhea americana* | Greater rhea | 1.5% | [17] | Feather mass |
| *Rhea pennata* | Lesser rhea | 1.8% | [17] | Feather mass |
| *Dromaius novaehollandiae* | Emu | 1.7% | [18] | Feather mass |

1. Brodkorb P (1955) Number of feathers and weights of various systems in a bald eagle. Wilson Bull 67: 142.

2. Daan S, Masman D, Groenewold A (1990) Avian basal metabolic rates: their association with body composition and energy expenditure in nature. Am J Physiol 259: 333–340.

3. DesRochers DW, Silbernagle MD, Nadig A, Reed JM (2010) Body Size, Growth, and Feather Mass of the Endangered Hawaiian Moorhen (*Gallinula chloropus sandvicensis*). Pacific Sci 64: 327–333. doi:10.2984/64.2.327.

4. Schorger AW (1966) The wild turkey. Its history and domestication. Norman: University of Oklahoma Press.

5. Leeson S, Walsh T (2010) Feathering in commercial poultry I. Feather growth and composition. Worlds Poult Sci J 60: 42–51. doi:10.1079/WPS20033.

6. Wolf P, Rabehl N, Kamphues J (2003) Investigations on feathering, feather growth and potential influences of nutrient supply on feathers’ regrowth in small pet birds (canaries, budgerigars and lovebirds). J Anim Physiol Anim Nutr 87: 134–141.

7. Summers RW, Underhill LG, Nicoll M, Rae R, Piersma T (1992) Seasonal, size- and age-related patterns in body-mass and composition of Purple Sandpipers *Calidris maritime* in Britain. Ibis 134: 346–354.

8. Wetmore A (1936) The number of contour feathers in Passeriform and related birds. Auk 53: 159–169.

9. Hopps EC (2002) Information on Waterfowl Feather Characteristics. Trans Illinois State Acad Sci 95: 229–237.

10. Cooper SJ (2002) Seasonal metabolic acclimatization in mountain chickadees and juniper titmice. Physiol Biochem Zool 75: 386–395. doi:10.1086/342256.

11. Swanson DL (1991) Seasonal adjustments in metabolism and insulation in the dark-eyed Junco. Condor 93: 538–545.

12. Klaassen M (1995) Moult and basal metabolic costs in males of two subspecies of stonechats: the European *Saxicola torquata rubicula* and the East African *S. t. axillaris*. Oecologia 104: 424–432.

13. Lill A, Box J, Baldwin J (2006) Do metabolism and contour plumage insulation vary in response to seasonal energy bottlenecks in superb fairy-wrens? Aust J Zool 54: 23–30. doi:10.1071/ZO05029.

14. Reid B, Williams G (1975) The Kiwi. In: Kuschel G, editor. Biogeography and Ecology in New Zealand. The Hague: Dr W. Junk b.v., Publishers. pp. 301–330.

15. Brand TS, Jordaan JW, Bhiya CS, Aucamp BB (2010) Effect of slaughter age and sex on the production output of South African Black ostriches. Br Poult Sci 51: 510–514. doi:10.1080/00071668.2010.502517.

16. Morris CA, Harris SD, May SG, Jackson TC, Hale DS, et al. (1995) Ostrich slaughter and fabrication: 1. Slaughter yields of carcasses and effects of electrical stimulation on post-mortem pH. Poult Sci 74: 1683–1687.

17. Sales J, Navarro JL, Bellis L, Manero a, Lizurume M, Martella MB (1997) Carcase and component yields of rheas. Br Poult Sci 38: 378–380. doi:10.1080/00071669708418006.

18. Sales J, Horbanczuk J, Dingle J, Coleman R, Sensik S (1999) Carcase characteristics of emus (*Dromaius novaehollandiae*). Br Poult Sci 40: 145–147.
